# Volume Alterations in Thalamic Subnuclei in Parkinson's Disease Dementia and Machine Learning‐Based Prediction of Diagnosis and Severity

**DOI:** 10.1002/brb3.71494

**Published:** 2026-06-25

**Authors:** Yingchuan Chen, Guanyu Zhu, Ruoyu Ma, Rujin Wang, Fangang Meng, Anchao Yang, Tingting Du, Jianguo Zhang

**Affiliations:** ^1^ Department of Neurosurgery, Beijing Tiantan Hospital Capital Medical University Beijing China; ^2^ Beijing Key Laboratory of Neurostimulation Beijing China; ^3^ Department of Functional Neurosurgery, Beijing Neurosurgical Institute Capital Medical University Beijing China

**Keywords:** dementia, machine learning, magnetic resonance imaging, Parkinson's disease

## Abstract

**Background:**

Cognitive decline in Parkinson's disease (PD) is associated with pathological alterations within the thalamus. Nevertheless, volumetric changes in the specific subnuclei of the thalamus in PD patients with dementia (PD‐D) remain inadequately characterized. Furthermore, the clinical challenges of diagnosing PD‐D at an individual level and forecasting the trajectory of cognitive decline persist.

**Methods:**

This study acquired structural magnetic resonance imaging (MRI) data from 60 healthy normal controls (NC), 63 PD patients without dementia (PD‐nD), and 57 PD‐D patients. The volumes of 25 thalamic subnuclei were quantified using FreeSurfer and a novel thalamic segmentation algorithm. Subsequently, individual PD‐D diagnosis and severity prediction of cognitive impairment were performed using support vector machines (SVMs).

**Results:**

Our findings demonstrated atrophy in seven out of 25 left and two out of 25 right thalamic subnuclei in PD‐D patients relative to PD‐nD patients. When compared to NC subjects, the PD‐D group exhibited volume reductions in two left and one right subnuclei, alongside enlargement in several others. Within the PD cohort, the volumes of four left thalamic subnuclei showed a negative correlation with cognitive impairment severity. Machine learning models achieved high accuracy in differentiating PD‐nD from NC (89.19%), PD‐D from NC (94.29%), and PD‐D from PD‐nD (83.33%). Moreover, the prediction of Mini‐Mental State Examination (MMSE) scores yielded a Pearson correlation coefficient of 0.7568.

**Conclusion:**

Specific thalamic subnuclei undergo atrophy in PD‐D, and these morphological changes are linked to cognitive deficit severity. Leveraging these features with machine learning enables accurate individual diagnosis and severity prediction.

## Graphical abstract

Structural MRI undergoes quality control, FreeSurfer‐based cortical reconstruction/volumetric segmentation, and thalamic subnuclei segmentation, followed by subnuclei volume comparison. The optimal feature set trains a model to predict cognitive impairment diagnosis/severity in a test set. Model performance is validated via permutation testing. Specific thalamic subnuclei undergo atrophy in PD‐D, and these morphological changes are linked to cognitive deficit severity. Leveraging these features with machine learning enables accurate individual diagnosis and severity prediction.

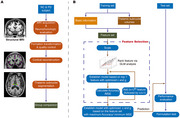

**Abbreviations**
ANCOVA: analysis of covariance
ANOVA: analysis of variance
AUC: area under curve
CM: centromedial nucleus
DTI: diffusion tensor imaging
EC: evolutionary computation
GA: genetic algorithm
GLM: general linear model
GM: gray matter
ICV: intracranial volume
LGN: lateral geniculate nucleus
L‐Sg: limitans (suprageniculate) nucleus
MDS: Movement Disorder Society
MMSE: Mini‐Mental State Examination
MPRAGE: magnetization‐prepared rapid acquisition gradient echo
MRI: magnetic resonance imaging
MSE: mean square error
NC: normal control
PD: Parkinson's disease
PD‐D: PD with dementia
PD‐nD: PD without dementia
Pf: parafascicular nucleus
Pt: paratenial nucleus
PuL: pulvinar lateral nucleus
ROC: receiver operating characteristic
SD: standard deviations
SVMs: support vector machines
UPDRS: United Parkinson Disease Rating Scale
VLa: ventral lateral anterior nucleus
VLp: ventral lateral posterior nucleus
VM: ventromedial nucleus
VPL: ventral posterolateral nucleus

## 1 Introduction

Parkinson's disease (PD) with dementia (PD‐D) is a decline in thinking, memory, and judgment that develops in individuals with an established diagnosis of PD, typically appearing at least 1 year after the onset of motor symptoms, which significantly diminishes quality of life and increases mortality risk (Blommer et al. 2023; Goetz, Emre, et al. 2008; Levy et al. 2002). Longitudinal data indicate that the cumulative incidence of dementia can reach 80% over the disease course (Gallagher et al. 2024; Hely et al. 2008). The prevalence of PD‐D is estimated to be between 25%–30%, a rate sixfold higher than in the general population (Kulisevsky and Pagonabarraga 2009), underscoring a strong link between PD pathology and cognitive decline.

Neuroimaging studies have associated PD‐D with morphological and connectivity changes throughout the brain. Gray matter (GM) atrophy, detectable via structural magnetic resonance imaging (MRI), is recognized as a marker of the neurodegenerative processes underlying cognitive impairment in PD (Borroni et al. 2015; Chen et al. 2024). White matter integrity is also compromised, with diffusion tensor imaging (DTI) revealing microstructural deterioration in PD‐D, characterized by significantly reduced fractional anisotropy in major tracts (Hattori et al. 2012).

The thalamus is a critical hub within the cortico‐basal ganglia‐thalamo‐cortical loop. Dysfunction of thalamic neuronal ensembles influences consciousness states and the perception of events, thoughts, and actions (Pifl et al. 2012). Reduced volumes of the thalamus were observed in PD with cognitive impairment (Mak et al. 2014). Given that the thalamus comprises numerous subnuclei with distinct functions, elucidating their specific morphological changes in PD‐D is crucial. However, this has been hampered by the historical lack of precise and reliable segmentation methods for these small structures. In a recent study, a Bayesian segmentation algorithm was developed for automated in vivo parcellation. Validations showed high test–retest reliability, robustness across MRI contrasts, and improved group discrimination in Alzheimer's disease compared with whole‐thalamus measures. Integrated into FreeSurfer, this method enables reliable, fine‐grained thalamic subnucleus analysis for neurodegenerative diseases, neurosurgery, and large‐scale imaging studies (Iglesias et al. 2018).

Diagnosis of PD‐D is a complex issue. Previously, a study achieved accuracy greater than 90% in distinguishing PD‐D from PD without dementia (PD‐nD) based on the differences in cortical thickness (Morales et al. 2013). However, diagnostic criteria of PD‐D were different from the recommendation of “Movement Disorder Society (MDS) Task Force” (Dubois et al. 2007). Additionally, the prediction of cognitive impairment severity in PD using neuroimaging biomarkers remains largely unexplored.

The present work aimed to elucidate the morphological alterations of thalamic subnuclei in PD‐D patients and their relationship with cognitive impairment severity. Furthermore, we sought to develop machine learning models based on thalamic subnuclei volumes for the individual diagnosis of PD‐D and the prediction of cognitive deficit severity.

## 2 Materials and Methods

### 2.1 Participants and Neuropsychology Evaluation

The study cohort was recruited from Beijing Tiantan Hospital, Capital Medical University, China, between October 2018 and August 2019. The Beijing Tiantan Hospital Ethics Committee approved the protocol (No. KY2016‐037‐02). All patients received a diagnosis of idiopathic PD with bilateral involvement from neurologists, fulfilling the UK Brain Bank criteria (Hughes et al. 1992). PD‐D classification was based on the Level I diagnostic criteria established by the MDS (Dubois et al. 2007). Control subjects had no history of PD or other neurological disorders. Motor severity was assessed using Part III of the MDS‐United Parkinson's Disease Rating Scale (UPDRS) in the OFF‐dopamine state (Goetz, Tilley, et al. 2008). Demographic information (age, sex, disease duration, education) and Mini‐Mental State Examination (MMSE) scores were collected. The final sample included 60 normal control (NC), 63 PD‐nD, and 57 PD‐D participants.

### 2.2 MRI Acquisition

All scans were performed on a 3.0 T Philips Ingenia MRI scanner (Philips Medical Systems, Best, The Netherlands) equipped with a 32‐channel head coil. Head motion was minimized using cushions. A high‐resolution T1‐weighted 3D magnetization‐prepared rapid acquisition gradient echo (MPRAGE) sequence was acquired for each participant (parameters: repetition time: 6.6 ms, echo time: 3.1 ms, flip angle: 8°, matrix size: 240 × 240, isotropic voxel: 1 mm^3^, slices: 196).

### 2.3 Image Processing

**FIGURE 1:**
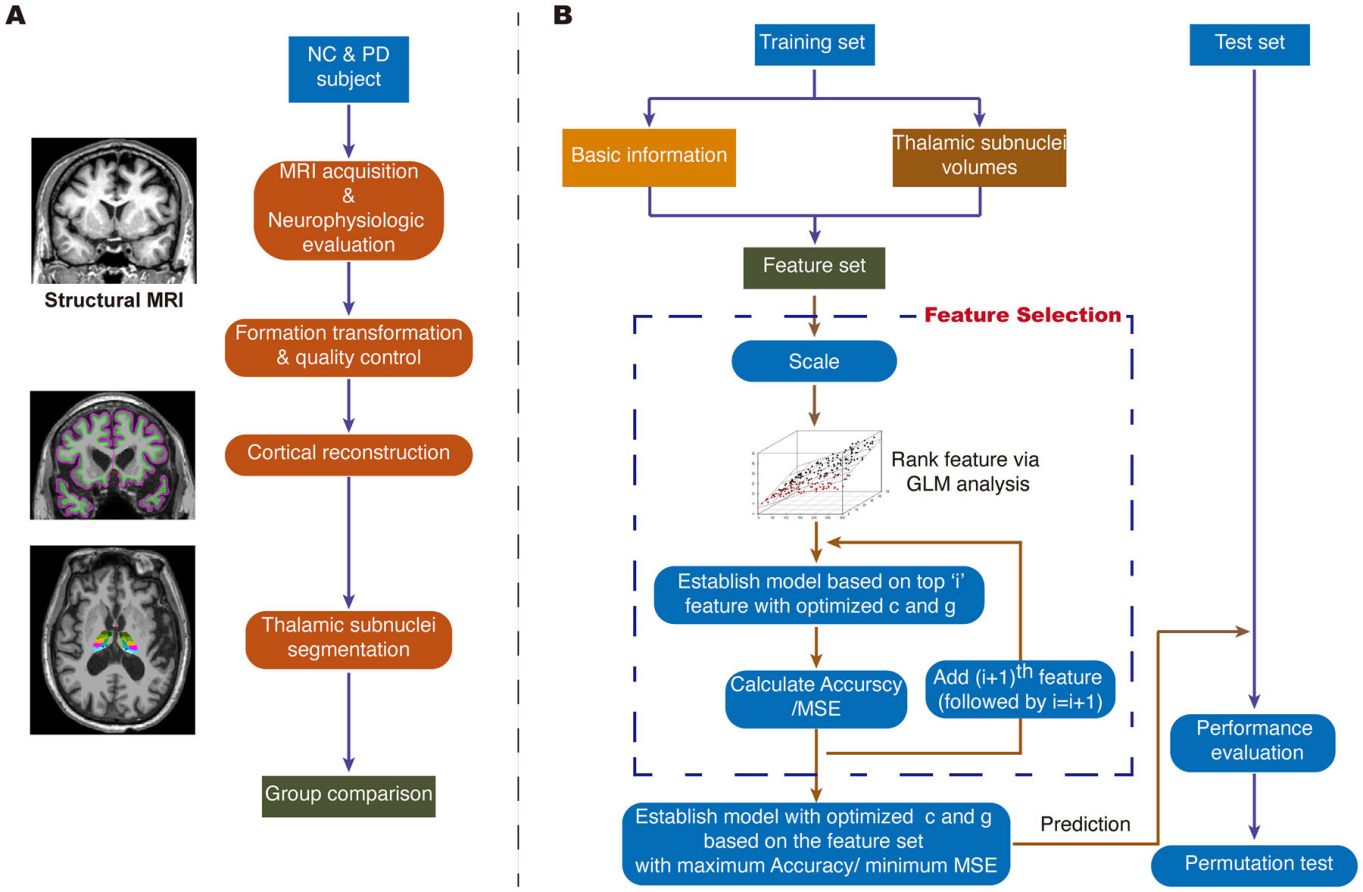
(A) The flow chart of volume evaluation and comparison of thalamic subnuclei. All DICOM files of structural MRI are converted into NifTi formats, and the quality of data were carefully checked. Image analysis was performed with FreeSurfer software to obtain primary cortical reconstruction and volumetric segmentation, then, was processed by thalamic segment algorithm. Finally, the volumes of each thalamic subnuclei were compared. (B) Flow chart of machine learning. All features of thalamus were ranked using the general liner model (GLM) based on *p* and beta value. The accuracy or mean square error (MSE) of cross validation in the top i feature was calculated, and the feature set with maximum accuracy or minimum MSE was selected and used to establish the final model. The model was then used to predict the diagnosis and severity of cognitive impairment in the test set. The performance of the machine learning was evaluated followed by permutation test.

DICOM files were converted to NifTi format using SPM12, followed by rigorous quality control for sharpness, coverage, motion, and orientation. Cortical reconstruction and volumetric segmentation were conducted with FreeSurfer software (development version) using standard pipelines (Fischl 2012). This process included non‐brain tissue removal, Talairach transformation, subcortical segmentation, intensity normalization, and surface deformation. The resulting files were subsequently processed with a dedicated thalamic nuclei segmentation algorithm (https://surfer.nmr.mgh.harvard.edu/fswiki/ThalamicNuclei) (Iglesias et al. 2018). This Bayesian procedure involves spatial warping of a probabilistic atlas, voxel‐wise segmentation, and intensity modeling. Finally, the volume of each thalamic subnucleus was extracted and normalized by the intracranial volume (ICV) (Jia et al. 2015) (Figure 1A).

### 2.4 Diagnosis and Severity of Cognitive Impairment Prediction

#### 2.4.1 Support Vector Machines (SVMs)

SVMs, a robust machine learning method known for its efficacy and computational efficiency in classification and regression tasks, were employed. Implementations were carried out using the LIBSVM package (version 3.2) within the MATLAB (version 2018b) environment (Chang and Lin 2011).

#### 2.4.2 Feature Selection

To enhance model performance and avoid overfitting, feature selection was conducted exclusively within the training set. Features were first standardized (*z*‐score). For classification tasks, a general linear model (GLM) was fitted to assess inter‐group differences while controlling for age and sex. Features were then ranked by their resulting *p*‐values in ascending order (Dong and Wang 2009). For the regression task (predicting MMSE), the GLM evaluated the association between each feature and the MMSE score (covarying for age and sex). Features were ranked by the absolute value of the *β*‐coefficient in descending order. The optimal feature set was defined as the one yielding the highest cross‐validation accuracy (classification) or the lowest mean square error (MSE) (regression) after hyperparameter optimization.

#### 2.4.3 Group Assignment, Hyperparameter Optimization, and Prediction

Participants were randomly stratified into training and test sets (Table S1) (Suomi et al. 2019). A genetic algorithm (GA), a type of evolutionary computation (EV), was utilized to optimize SVM hyperparameters, maximizing model efficacy (Zeng et al. 2018). A fivefold cross‐validation procedure within the training set was used for this optimization (Wilkes et al. 2018). The final model, trained on the entire training set with the optimized hyperparameters, was evaluated on the held‐out test set (Figure 1B). SVM classification was used for group differentiation, and SVM regression was applied to predict MMSE scores in PD patients.

### 2.5 Performance of Machine Learning Evaluation

For classification, according to the previous study (Salvatore et al. 2014), the accuracy, specificity, and sensitivity were calculated as follows:

**Table:** 

		Real	
		Negative	Positive
Predict	Negative	True negative	False negative
	Positive	False positive	True positive

Accuracy = (True negative + True positive)/(True negative + True positive + False positive + False negative)

Sensitivity = True positive/(False negative + True positive)

Specificity = True negative/(True negative + False positive)

For regression, Pearson correlation coefficient (*r*), coefficient of determination (*R* ^2^), and AUC (area under curve) of receiver operating characteristic curve (ROC) were defined and computed as in the previous studies (Nakagawa et al. 2017).

A permutation test (2000 iterations) was performed to assess the statistical significance of the results against chance. For each iteration, labels were randomly shuffled, and the entire pipeline (feature selection, hyperparameter optimization, prediction) was repeated. The *p*‐value was derived from the proportion of iterations where the permuted‐label performance exceeded the real‐label performance (Gould et al. 2014).

### 2.6 Statistical Analysis

Data are presented as mean ± standard deviation (SD). Group differences in clinical data were assessed using two‐sample *t*‐tests, Mann–Whitney *U* tests, one‐way analysis of variance (ANOVA), or chi‐squared tests, as appropriate. Analysis of covariance (ANCOVA), controlling for age and sex, was used to evaluate group differences in subnuclei volumes. Post‐hoc analyses employed Bonferroni correction for multiple comparisons. Statistical analyses and plotting were conducted in MATLAB, with a significance threshold of *p* < 0.05.

## 3 Results

### 3.1 Participant Characteristics

The study included 60 NC, 63 PD‐nD, and 57 PD‐D participants. The three groups were well‐matched for age, sex, and years of education. Disease duration and Hoehn and Yahr stage did not differ significantly between the PD‐nD and PD‐D groups. As expected, PD‐D patients exhibited significantly lower MMSE scores and more severe motor impairment (higher MDS‐UPDRS III scores), consistent with prior literature (Morales et al. 2013). Details are provided in Table 1.

**TABLE 1:** Characteristics of people in different groups.

	NC (*n* = 60)	PD‐nD (*n* = 63)	PD‐D (*n* = 57)	*p* value
Sex (male/female)	31/29	32/31	27/30	0.8867
Age (years)	61.8 ± 5.9	62.4 ± 7.9	63.9 ± 9.0	0.0686
MDS‐UPDRS III score	—	46.0 ± 14.9	54.6 ± 18.2	0.0057
Disease duration (years)	—	8.7 ± 4.4	9.4 ± 4.5	0.3834
Hoehn and Yahr stage (median [25%, 75%])	—	3 (3, 3)	3 (3, 3)	0.3474
Education year (years)	12.4 ± 3.2	12.1 ± 3.1	11.1 ± 3.4	0.0851
MMSE score	—	28.3 ± 1.2	21.6 ± 3.4	0.0000

Abbreviations: MDS‐UPDRS, Movement Disorder Society‐United Parkinson Disease Rating Scale; MMSE, Mini‐Mental State Examination; NC, normal control; PD, Parkinson's disease; PD‐D, PD with dementia; PD‐nD, PD without dementia.

### 3.2 Thalamic Subnuclei Atrophy in PD‐D

Volumes of thalamic subnuclei, corrected for ICV, age, and sex, were compared across groups, with Bonferroni correction applied.

**FIGURE 2:**
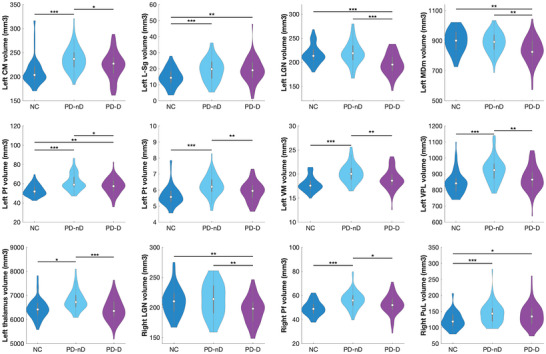
The violin plots of thalamic subnuclei volume in different groups. Compared with NC subjects, the left centromedial nucleus (CM), limitans (suprageniculate) nucleus (L‐Sg), parafascicular nucleus (Pf), paratenial nucleus (Pt), ventromedial nucleus (VM), ventral posterolateral nucleus (VPL), thalamus, right Pf, and pulvinar lateral nucleus (PuL) were significantly enlarged in PD‐nD patients. A notable reduction in volumes of left CM, lateral geniculate nucleus (LGN), MDm, Pf, Pt, VM, VPL, thalamus, right LGN, and Pf were in PD‐D patients by comparison with those of PD‐nD patients. Moreover, compared with the thalamic subnuclei of NC people, the left L‐Sg, Pf, and right PuL were obviously enlarged in PD‐D patients. However, the left LGN, MDm, and right LGN were atrophied in PD‐D patients, compared with those of NC people. NC, normal control; PD, Parkinson's disease; PD‐D, PD with dementia; PD‐nD, PD without dementia. ^*^ *p* < 0.05; ^**^ *p* < 0.01; ^***^ *p* < 0.001.

Compared to NC, PD‐nD patients showed significant volume enlargement in several left‐sided nuclei (centromedial nucleus [CM], limitans [suprageniculate] nucleus [L‐Sg], parafascicular nucleus [Pf], paratenial nucleus [Pt], ventromedial nucleus [VM], ventral posterolateral nucleus [VPL]) and the left whole thalamus, as well as in the right Pf and pulvinar lateral nucleus (PuL) (Figure 2 and Table 2).

**TABLE 2:** The volume of thalamic subnuclei in the different groups.

Nuclei	NC (mm^3^)^a^	PD‐nD (mm^3^)^a^	PD‐D (mm^3^)^a^	*p* (NC vs. PD‐nD)^b^	*p* (NC vs. PD‐D)^b^	*p* (PD‐nD vs. PD‐D)^b^
**Left hemisphere**						
CM	108.0 ± 16.7	112.8 ± 20.0	107.4 ± 22.3	0.0000	> 0.05	0.0260
L‐Sg	14.6 ± 5.5	19.3 ± 6.9	18.9 ± 7.6	0.0005	0.0020	> 0.5
LGN	215.9 ± 21.5	218.7 ± 24.1	196.0 ± 21.4	> 0.05	0.0000	0.0000
MDm	898.5 ± 74.3	888.3 ± 68.8	834.8 ± 85.3	> 0.05	0.0000	0.0005
Pf	53.1 ± 5.7	61.7 ± 8.7	57.5 ± 8.4	0.0000	0.0061	0.0121
Pt	5.7 ± 0.6	6.3 ± 0.7	5.9 ± 0.6	0.0000	> 0.05	0.0091
VM	18.3 ± 3.0	20.2 ± 1.9	18.9 ± 2.1	0.0000	> 0.05	0.0081
VPL	861.0 ± 123.9	926.0 ± 77.2	870.6 ± 85.7	0.0008	> 0.05	0.0067
Whole thalamus	6530.9 ± 775.5	6792.0 ± 409.4	6404.7 ± 453.7	0.0357	> 0.05	0.0008
**Right hemisphere**						
LGN	212.1 ± 28.6	212.4 ± 26.8	195.6 ± 22.0	> 0.05	0.0023	0.0016
Pf	49.4 ± 5.9	56.2 ± 7.3	52.4 ± 8.0	0.0000	> 0.05	0.0117
PuL	122.3 ± 26.0	143.9 ± 32.1	138.6 ± 34.6	0.0005	0.0152	> 0.05

Abbreviations: CM, centromedial nucleus; ICV, intracranial volume; L‐Sg, limitans (suprageniculate) nucleus; MDm, mediodorsal medial magnocellular nucleus; NC, normal control; PD, Parkinson's disease; PD‐D, PD with dementia; PD‐nD, PD without dementia; Pf, parafascicular nucleus; Pt, paratenial nucleus; PuL, pulvinar lateral nucleus; VM, ventromedial nucleus; VPL, ventral posterolateral nucleus.

^a^ Corrected by ICV, age, and sex.

^b^ *p* value with Bonferroni correction.

In contrast, PD‐D patients demonstrated significant volume reduction in multiple left‐sided nuclei (CM, lateral geniculate nucleus [LGN], mediodorsal medial magnocellular nucleus [MDm], Pf, Pt, VM, VPL, whole thalamus) and the right LGN and Pf when compared to PD‐nD patients. Relative to NC, the PD‐D group showed enlargement in the left L‐Sg and Pf and the right PuL, but atrophy in the left LGN and MDm and the right LGN (Figure 2 and Table 2).

### 3.3 Association Between Thalamic Subnuclei and Severity of Cognitive Impairment

The MMSE score is an index that reflects the severity of cognitive impairment (Perneczky et al. 2006). Analysis across all PD patients revealed that the severity of cognitive impairment (MMSE score) was significantly associated with volume loss in several left thalamic structures: the LGN, MDm, ventral lateral anterior nucleus (VLa), ventral lateral posterior nucleus (VLp), and the whole thalamus. No significant associations were found for right thalamic subnuclei. Details are shown in Table 3.

**TABLE 3:** The association between MMSE score and thalamic subnuclei volume.

Nuclei	Beta	Standard beta	*p* (Bonferroni‐corrected)
**Left hemisphere**			
LGN	0.0667	1.8438	0.0002
MDm	0.0171	1.6031	0.0106
VLa	0.0250	1.3747	0.0330
VLp	0.0205	1.4711	0.0176
Whole thalamus	0.0033	1.6849	0.0015

Abbreviations: LGN, lateral geniculate nucleus; MDm, mediodorsal medial magnocellular nucleus; MMSE, Mini‐Mental State Examination; VLa, ventral lateral anterior nucleus; VLp, ventral lateral posterior nucleus.

### 3.4 Diagnostic Prediction of PD‐D via Machine Learning

**FIGURE 3:**
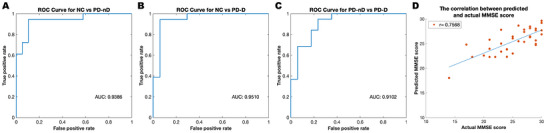
(A–C) The performance of machine learning classification. The accuracies of distinguishing PD‐nD from NC, PD‐D from NC, and PD‐D from PD‐nD were 89.19% (AUC: 0.9386), 94.29% (AUC: 0.9510), and 83.33% (AUC: 0.9102) respectively via machine learning. (D) The performance of machine learning regression. The performance of MMSE score prediction was achieved with Pearson correlation coefficient (*r*) of 0.7568 and coefficient of determination of 0.5725. MMSE, Mini‐Mental State Examination; NC, normal control; PD, Parkinson disease; PD‐D, PD with dementia; PD‐nD, PD without dementia.

Classifications of NC, PD‐nD, and PD‐D were carried out based on basic information (age and sex) and volumes of thalamic subnuclei with feature selection. In distinguishing PD‐nD from NC, an accuracy of 89.19%, with a sensitivity of 89.47% and specificity of 88.89% was achieved. In ROC analysis, an AUC of 0.9386 was obtained. For classification of PD‐D versus NC, accuracy, sensitivity, and specificity were 94.29%, 94.12%, and 94.44%, respectively. Furthermore, ROC analysis was performed, and an AUC value of 0.9510 was obtained. Finally, we tried to differentiate the PD‐D from PD‐nD, and attained an acceptable accuracy of 83.33%, with sensitivity of 82.35%, specificity of 84.21%, and AUC of 0.9102 (Figure 3A–C and Table 4).

**TABLE 4:** The performance of machine learning.

		Classification		Regression
	NC vs. PD‐nD	NC vs. PD‐D	PD‐nD vs. PD‐D	MMSE score
Accuracy	89.19%	94.29%	83.33%	—
Sensitivity	89.47%	94.12%	82.35%	—
Specificity	88.89%	94.44%	84.21%	—
AUC	0.9386	0.9510	0.9102	—
*r*	—	—	—	0.7568
*p* (of *r*)^a^	—	—	—	0.0000
*R* ^2^	—	—	—	0.5725
MSE	—	—	—	7.12
*p* (permut.)^b^	0.0000	0.0000	0.0000	0.0000

Abbreviations: AUC, area under curve; MMSE, Mini‐Mental State Examination; MSE, mean square error; NC, normal control; PD, Parkinson's disease; PD‐D, PD with dementia; PD‐nD, PD without dementia; *r*, Pearson correlation coefficient; *R* ^2^, coefficient of determination.

^a^ *p* value of Pearson correlation coefficient.

^b^ *p* value of permutation test.

Permutation tests confirmed that these results were highly significant (all *p* < 0.001), indicating performance well above chance levels (Figure S1A–C and Table 4). Model details are available in Table S2.

### 3.5 Prediction of Cognitive Impairment Severity via Machine Learning

The SVM regression model trained to predict MMSE scores in PD patients demonstrated strong performance on the test set, with a Pearson correlation of *r* = 0.7568 (*R* ^2^ = 0.5725) between the predicted and actual scores (Figure 3D). The permutation test for this regression model was also significant (Figure S1D and Table 4). The details of the regression model are shown in Table S2.

## 4 Discussion

To the best of our knowledge, this represents the first in vivo investigation of volumetric changes across thalamic subnuclei in PD‐D patients and their association with cognitive impairment severity. Our results demonstrate significant volumetric differences in specific nuclei among NC, PD‐nD, and PD‐D groups. Furthermore, we show that machine learning models leveraging these thalamic features can achieve accurate individual diagnosis of PD‐D and prediction of cognitive deficit severity.

### 4.1 Thalamic Subnuclei Atrophy in PD‐D

Prior research has established that cortical atrophy in regions like the frontal and entorhinal cortex is associated with cognitive impairment in PD (Borroni et al. 2015; Bouwman et al. 2025; Jia et al. 2019), highlighting GM loss as a key pathological component of PD‐D. Subcortical structures, including the hippocampus and thalamus, also exhibit volume loss in PD‐D (Mak et al. 2014; Tanner et al. 2017).

Postmortem evidence indicates significantly lower overall thalamic D_2_ receptor density in PD‐D compared to PD‐nD, with notable variations across specific subnuclei (Piggott et al. 2007). Previously, a study identified PD with mild cognitive impairment as converters or nonconverters, depending on whether they were subsequently diagnosed as PD‐D. Subcortical shape analysis revealed that PD with mild cognitive impairment converters had smaller local shape volumes than nonconverters in the bilateral thalamus. Logistic regression analysis showed that local shape volumes in the bilateral thalamus were significantly independent predictors of PD with mild cognitive impairment converters (Chung et al. 2017). Functionally, cerebellar‐recipient neurons in the motor thalamus are critical for movement initiation (Prevosto and Sommer 2013).

Our study provides a detailed map of these changes, revealing bilateral subnuclei atrophy in PD‐D compared to PD‐nD, with several left‐sided nuclei correlating with cognitive severity. We employed rigorous statistical corrections for multiple comparisons. While PD‐D patients had greater motor impairment, consistent with literature (Morales et al. 2013; Poewe et al. 2008), we followed precedents (Apostolova et al. 2010; Foo et al. 2017) by not correcting for UPDRS‐III in group comparisons to avoid overadjustment. Interestingly, the observed thalamic enlargement in PD‐nD patients aligns with previous work (Jia et al. 2015), potentially reflecting compensatory gliosis or inflammatory processes in early disease stages. We hypothesize that initial thalamic enlargement may be followed by a distinct atrophic pathology as cognitive impairment emerges, a trajectory that warrants longitudinal investigation. In patients with PD‐D, neuronal or other cellular loss may occur in the thalamic subnuclei, which results in a certain degree of reduction in the volume of these nuclei. Under the influence of the aforementioned factors, the phenomenon of increased nuclear volume observed in PD‐nD patients is not prominent in PDD patients. A few studies support our hypothesis to some degree (Beheshti et al. 2024; Park et al. 2020; Yun et al. 2025).

### 4.2 Individual Diagnosis and Severity Prediction via Machine Learning

Machine learning is increasingly applied to PD diagnosis. Studies using whole‐brain GM thickness have reported accuracies of 65.7%–85.8% for distinguishing PD from controls (Huppertz et al. 2016; Salvatore et al. 2014). However, individual diagnosis of PD‐D and quantitative prediction of cognitive severity remain challenging, with few machine learning studies addressing them. While one study achieved > 90% accuracy for PD‐D classification using cortical thickness (Morales et al. 2013), its small sample and diagnostic criteria limit generalizability.

Our study implemented a rigorous machine learning pipeline. Feature selection was confined to the training set to prevent information leakage and ensure a non‐overoptimistic performance estimate. Given the sample size, we used cross‐validation for hyperparameter optimization, a well‐established practice (Douglas et al. 2011; Wilkes et al. 2018). Our results strongly suggest that thalamic subnuclei morphology holds significant potential as a biomarker for the individual diagnosis of PD‐D and the prediction of cognitive impairment severity.

## 5 Conclusion

This is the first “in vivo” study to detail morphological changes in thalamic subnuclei in PD‐D patients and to employ machine learning for predicting cognitive impairment severity. We found atrophy in seven left and two right thalamic subnuclei in PD‐D compared to PD‐nD. Relative to NC, PD‐D showed a complex pattern of both volume reduction (two left, one right subnuclei) and enlargement. The volumes of four left thalamic subnuclei were negatively correlated with cognitive deficit severity. Machine learning models achieved high accuracy in diagnostic classification (83.33%–94.29%) and robust MMSE score prediction (*r* = 0.7568, *R* ^2^ = 0.5725). These findings underscore the role of thalamic subnuclei in PD‐D pathology and their utility as quantitative neuroimaging biomarkers.

## Author Contributions

Guanyu Zhu: methodology. Anchao Yang: writing – original draft. Rujin Wang: software. **Yingchuan Chen**: investigation. **Jianguo Zhang**: writing – review and editing, writing – original draft. **Tingting Du**: visualization, conceptualization. Fangang Meng: writing – review and editing. **Ruoyu Ma**: software.

This work was supported by the Beijing Natural Science Foundation (7254306) and the Beijing Municipal Science & Technology Commission and Administrative Commission of Zhongguancun Science Park (Z241100009024019).

## Conflicts of Interest

The authors declare no conflicts of interest.

## Supporting information




**Supplementary Tables**: brb371494‐sup‐0001‐Tables.docx

**Supplementary Figure 1. A‐D**. The permutation test of machine learning. The performances of machine learning on diagnosis and severity of cognitive impairment prediction were not by chance (All p < 0.001). NC, normal control; PD, Parkinson disease; PD‐nD, PD without dementia; PD‐D, PD with dementia; MMSE, Mini‐Mental State Examination; R^2^, coefficient of determination.

## Data Availability

Data will be available on request to the authors.
