# Supplementary material for: Volume Alterations in Thalamic Subnuclei in Parkinson's Disease Dementia and Machine Learning‐Based Prediction of Diagnosis and Severity
Source: Brain Behav. 2026 Jun 25;16(6):e71494. doi: 10.1002/brb3.71494 (PMC13297024; doi:10.1002/brb3.71494)
Supplement: Supplementary file 1 — Supplementary Tables: brb371494‐sup‐0001‐Tables.docx [file BRB3-16-e71494-s001.docx]

**Supplementary Table 1.** Characteristics of training set and test set

|  | **Sex (male/female)** | **Age (years)** | **MMSE score** | **MDS-UPDRS** | **Disease duration (years)** |
| --- | --- | --- | --- | --- | --- |
| **NC** |  |  |  |  |  |
| Training set | 22/20 | 61.6 ± 5.6 | - | - | - |
| Test set | 9/9 | 62.3 ± 7.3 | - | - | - |
| P value | 0.8657 | 0.6689 | - | - | - |
| **PD-nD** |  |  |  |  |  |
| Training set | 22/22 | 62.3 ± 7.8 | 28.3 ± 1.2 | 45.8 ± 13.8 | 9.2 ± 4.3 |
| Test set | 10/9 | 62.6 ± 8.1 | 28.3 ± 1.3 | 46.4 ± 17.4 | 7.4 ± 4.3 |
| P value | 0.8479 | 0.8940 | 0.9529 | 0.8883 | 0.1343 |
| **PD-D** |  |  |  |  |  |
| Training set | 19/21 | 64.3 ± 9.8 | 21.6 ± 3.6 | 53.2 ± 19.5 | 9.7 ± 4.8 |
| Test set | 8/9 | 66.5 ± 6.5 | 31.8 ± 3.1 | 58.0 ± 18.5 | 8.6 ± 3.6 |
| P value | 0.9757 | 0.4081 | 0.8704 | 0.378 | 0.4095 |

NC, Normal control; PD, Parkinson disease; PD-nD, PD without dementia; PD-D, PD without dementia; MDS-UPDRS, Movement Disorder Society-United Parkinson Disease Rating Scale; MMSE, Mini-Mental State Examination.

**Supplementary Table 2.** The features and hyperparameters in machine learning

|  | **Features** | **Cost** | **Gamma** | |
| --- | --- | --- | --- | --- |
| **Classification** |  |  | |  |
| NC vs PD-nD | Age, sex, Left PuM, Left MV(Re), Left L-Sg, Right AV, Left LD, Right LP, Right L-Sg, Left PuA, Right VM, Right VA, Left PuI, Right PuL, Left VPL, Left CL, Left CM, Right PuM, Right LGN, Left VAmc, Right VAmc, Right LD, Right PuI, Right Pf, Left Whole thalamus, Left MDl, Left Pf, Left VM, Left LGN, Left PuL, Left MDm, Right CeM, Left LP, Right Pc, Right Whole thalamus, Right MGN, Left Pt, Right PuA, Right MDl, Right VPL, Right Pt, Left AV, Left Pc and Right CM | 3.3 | 1.1 | |
| NC vs PD-D | Age, sex, Right CM, Right CL, Left MV(Re), Right PuI, Left MDm, Right VA, Left AV, Right Pf, Left LGN, Left LD, Right AV, Right VPL, Right VLp, Left CM, Left Pf and Left PuA | 3.0 | 3.3 | |
| PD-nD vs PD-D | Age, sex, Left Whole thalamus, Right L-Sg, Right CeM, Right Vamc, Right PuM, Right MV(Re), Left AV, Right MDl, Left Pt, Left MGN, Left PuL, Right Vla, Right LP, Left PuM and Left VA | 1.8 | 0.1 | |
| **Regression** |  |  | |  |
| MMSE score | Age, sex, Left VA, Right Pf, Right CeM, Right LD, Right VA, Right PuI, Left AV, Right LP, Left LGN, Left CL, Left Vamc, Right MDm, Right AV, Left Pt and Left Vla | 8.5 | 0.1 | |

NC, Normal control; PD, Parkinson disease; PD-nD, PD without dementia; PD-D, PD with dementia; MMSE, Mini-Mental State Examination; AV, anteroventral nucleus; LD, laterodorsal nucleus; LP, lateral posterior nucleus; VA, ventral anterior nucleus; VAmc, ventral anterior magnocellular nucleus; VLa, ventral lateral anterior nucleus; VLp, ventral lateral posterior nucleus; VPL, ventral posterolateral nucleus; VM, ventromedial nucleus; CeM, central medial nucleus; CL, central lateral nucleus; Pc, paracentral nucleus; CM, centromedial nucleus, Pf, parafascicular nucleus; Pt, paratenial nucleus; MV-re, reuniens (medial ventral) nucleus; MDm, mediodorsal medial magnocellular nucleus; MDl, mediodorsal lateral parvocellular nucleus; LGN, lateral geniculate nucleus; MGN, medial geniculate nucleus; L-SG, limitans (suprageniculate) nucleus; PuA, pulvinar anterior nucleus; PuM, pulvinar medial nucleus; PuL, pulvinar lateral nucleus; PuI, pulvinar inferior nucleus.
