# Supplementary figures and images for: Volume Alterations in Thalamic Subnuclei in Parkinson's Disease Dementia and Machine Learning‐Based Prediction of Diagnosis and Severity
Source: Brain Behav. 2026 Jun 25;16(6):e71494. doi: 10.1002/brb3.71494 (PMC13297024; doi:10.1002/brb3.71494)

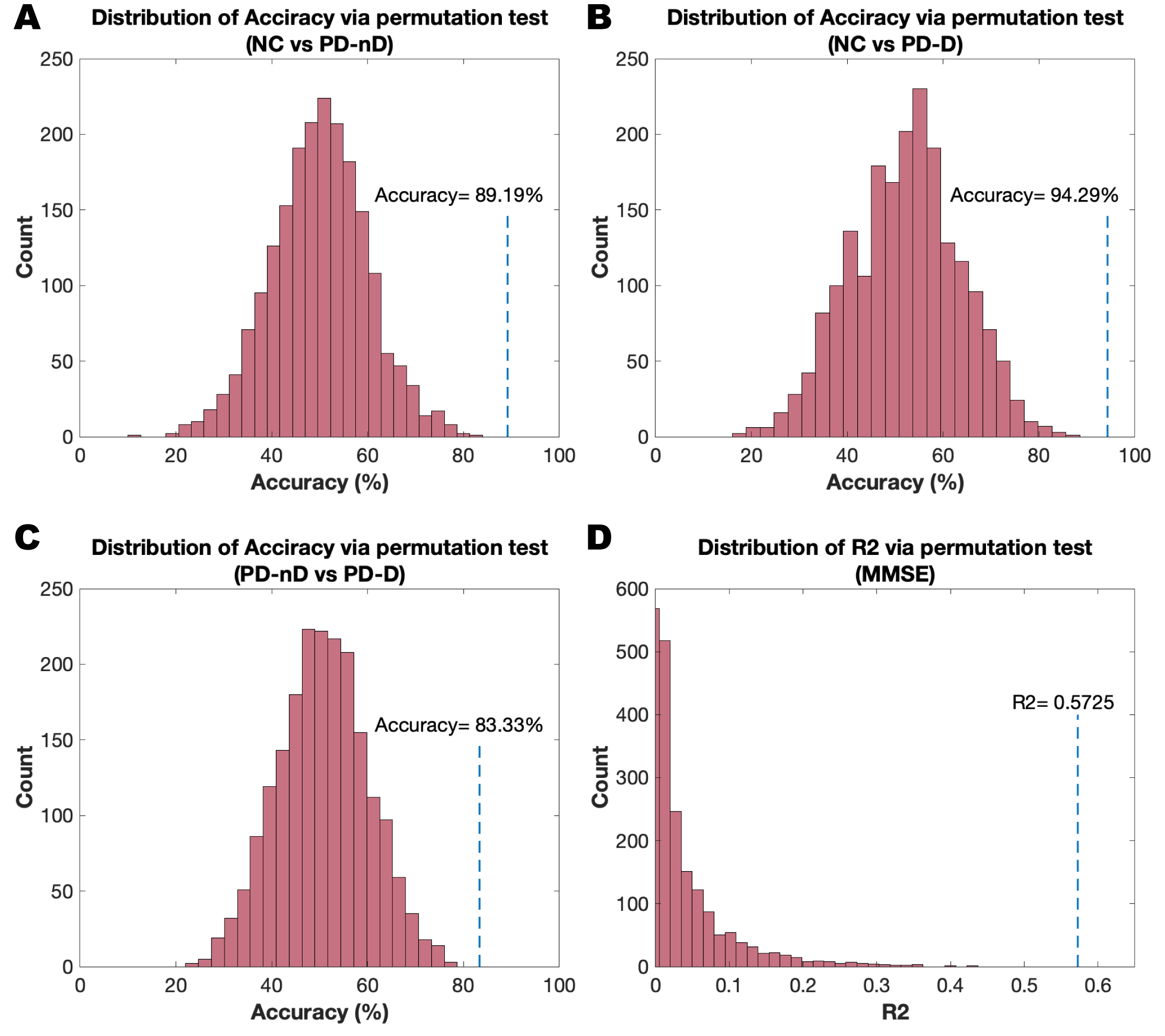

Supplement: Supplementary file 2 — Supplementary Figure 1. A‐D. The permutation test of machine learning. The performances of machine learning on diagnosis and severity of cognitive impairment prediction were not by chance (All p < 0.001). NC, normal control; PD, Parkinson disease; PD‐nD, PD without dementia; PD‐D, PD with dementia; MMSE, Mini‐Mental State Examination; R2, coefficient of determination. [file BRB3-16-e71494-s002.tif]
